# Impact of antiviral treatment and hospital admission delay on risk of death associated with 2009 A/H1N1 pandemic influenza in Mexico

**DOI:** 10.1186/1471-2334-12-97

**Published:** 2012-04-20

**Authors:** Gerardo Chowell, Cécile Viboud, Lone Simonsen, Mark A Miller, Santiago Echevarría-Zuno, Margot González-León, Víctor H Borja Aburto

**Affiliations:** 1 Mathematical, Computational & Modeling Sciences Center, School of Human Evolution and Social Change, Arizona State University, Tempe, AZ, USA; 2 Division of Epidemiology and Population Studies, Fogarty International Center, National Institutes of Health, Bethesda, MD, USA; 3 Department of Global Health, School of Public Health and Health Services, George Washington University, Washington DC, USA; 4 Dirección de Prestaciones Médicas, Instituto Mexicano del Seguro Social, Mexico City, Mexico; 5 Coordinación de Vigilancia Epidemiológica y Apoyo en Contingencias, Instituto Mexicano del Seguro Social, Mier y Pesado 120, México, DF 03100, Mexico; 6 Division of Epidemiology and Population Studies, Fogarty International Center, National Institutes of Health, School of Human Evolution and Social Change, Arizona State University, Bethesda, MD, USA

**Keywords:** 2009 A/H1N1 influenza pandemic, Neuraminidase inhibitors, Antivirals, Case fatality ratio, Multivariate logistic regression, Hospital admission delay, Pandemic wave, Mexico.

## Abstract

**Background:**

Increasing our understanding of the factors affecting the severity of the 2009 A/H1N1 influenza pandemic in different regions of the world could lead to improved clinical practice and mitigation strategies for future influenza pandemics. Even though a number of studies have shed light into the risk factors associated with severe outcomes of 2009 A/H1N1 influenza infections in different populations (e.g., [1-5]), analyses of the determinants of mortality risk spanning multiple pandemic waves and geographic regions are scarce. Between-country differences in the mortality burden of the 2009 pandemic could be linked to differences in influenza case management, underlying population health, or intrinsic differences in disease transmission [6]. Additional studies elucidating the determinants of disease severity globally are warranted to guide prevention efforts in future influenza pandemics.

In Mexico, the 2009 A/H1N1 influenza pandemic was characterized by a three-wave pattern occurring in the spring, summer, and fall of 2009 with substantial geographical heterogeneity [7]. A recent study suggests that Mexico experienced high excess mortality burden during the 2009 A/H1N1 influenza pandemic relative to other countries [6]. However, an assessment of potential factors that contributed to the relatively high pandemic death toll in Mexico are lacking. Here, we fill this gap by analyzing a large series of laboratory-confirmed A/H1N1 influenza cases, hospitalizations, and deaths monitored by the Mexican Social Security medical system during April 1 through December 31, 2009 in Mexico. In particular, we quantify the association between disease severity, hospital admission delays, and neuraminidase inhibitor use by demographic characteristics, pandemic wave, and geographic regions of Mexico.

**Methods:**

We analyzed a large series of laboratory-confirmed pandemic A/H1N1 influenza cases from a prospective surveillance system maintained by the Mexican Social Security system, April-December 2009. We considered a spectrum of disease severity encompassing outpatient visits, hospitalizations, and deaths, and recorded demographic and geographic information on individual patients. We assessed the impact of neuraminidase inhibitor treatment and hospital admission delay (≤ > 2 days after disease onset) on the risk of death by multivariate logistic regression.

**Results:**

Approximately 50% of all A/H1N1-positive patients received antiviral medication during the Spring and Summer 2009 pandemic waves in Mexico while only 9% of A/H1N1 cases received antiviral medications during the fall wave (P < 0.0001). After adjustment for age, gender, and geography, antiviral treatment significantly reduced the risk of death (OR = 0.52 (95% CI: 0.30, 0.90)) while longer hospital admission delays increased the risk of death by 2.8-fold (95% CI: 2.25, 3.41).

**Conclusions:**

Our findings underscore the potential impact of decreasing admission delays and increasing antiviral use to mitigate the mortality burden of future influenza pandemics.

## Background

Increasing our understanding of the factors affecting the severity of the 2009 A/H1N1 influenza pandemic in different regions of the world could lead to improved clinical practice and mitigation strategies for future influenza pandemics. Even though a number of studies have shed light into the risk factors associated with severe outcomes of 2009 A/H1N1 influenza infections in different populations (e.g., [1-5]), analyses of the case fatality ratio, e.g. the proportion of deaths among all symptomatic infections, spanning multiple pandemic waves, and geographic regions are scarce. Between-country differences in the mortality burden of the 2009 pandemic [6] could be linked to differences in influenza case management, underlying population health, or intrinsic differences in disease transmission. Additional studies elucidating the determinants of disease severity globally are warranted to guide prevention efforts in future influenza pandemics.

In Mexico, the 2009 A/H1N1 influenza pandemic was characterized by a three-wave pattern occurring in the spring, summer, and fall of 2009 with substantial geographical heterogeneity [7]. A recent study suggests that Mexico experienced high excess mortality burden during the 2009 A/H1N1 influenza pandemic relative to other countries [6]. However, an assessment of potential factors that contributed to the relatively high pandemic death toll in Mexico are lacking. Here, we fill this gap by analyzing a large series of laboratory-confirmed A/H1N1 influenza cases, hospitalization, and deaths monitored by the Mexican Social Security medical system during April 1 through December 31, 2009 in Mexico. In particular, we quantify the association between disease severity, hospital admission delays, and neuraminidase inhibitor use by demographic information, pandemic wave, and geographic regions of Mexico.

## Methods

### Epidemiological data

We used patient level data collected by a prospective epidemiological surveillance system put in place specifically for the 2009 influenza pandemic by the Mexican Institute for Social Security (IMSS) [7,8]. IMSS is a tripartite Mexican health system covering approximately 40% of the Mexican population comprising workers in the private sector and their families, relying on a network of 1,099 primary health-care units and 259 hospitals nationwide. The age and gender distributions of the population affiliated with IMSS are representative of the general Mexican population [7].

We obtained information on all patients attending any primary-care clinic or hospital with influenza-like-illness (ILI) reported across 32 Mexican states between April 1 and December 31, 2009. Patient-level information was entered into a standardized online form by hospital or clinic epidemiologists during the pandemic; a single record was obtained for each patient, ensuring that each patient is counted only once. ILI was defined as a combination of cough, headache, fever, and one or more of the following symptoms: sore throat, rhinorrhoea, arthralgias, myalgia, prostration, thoracic pain, abdominal pain, nasal congestion, diarrhea. For persons > 65 years presence of fever was not required [8], while for infants, irritability was included in the list of associated symptoms.

Respiratory swabs were obtained from ILI patients for influenza testing [7]; rRT-PCR was performed to identify influenza A/H1N1 [9] by the Instituto de Diagnóstico y Referencia Epidemiológica (InDRE) until May 25, 2009, after which point samples were analyzed by La Raza, an IMSS auxiliary laboratory to InDRE. The proportion of ILI patients tested for influenza increased rapidly during the first 2–3 weeks of the pandemic, at which point testing rates remained stable at ~33% throughout the pandemic, across geographic regions, and age groups [7].

For each ILI patient, we compiled demographic information (age in yrs, and gender), pandemic A/H1N1 status (positive, negative; for those tested), disease severity (outpatient, inpatient, and death), reporting state (including 31 states plus the Federal District), dates of onset of symptoms (self-reported) and hospital admission and discharge (if hospitalized), and whether the patient was treated with neuraminidase inhibitors upon initial consultation or at hospital admission. Hospitalized patients had to be admitted with acute respiratory infection (ARI), defined as respiratory difficulty with fever and cough, combined with one or more of the following clinical symptoms: confinement to bed, thoracic pain, polypnea, or acute respiratory distress syndrome. Children <5 years with pneumonia or severe pneumonia that required hospitalization were also considered as ARI cases.

In Mexico, antiviral treatment with neuraminidase inhibitors (Oseltamivir and Zanamivir) was considered for all ILI cases upon initial clinical evaluation and individual risk of developing complications [10]. Specifically, antiviral treatment was recommended for all cases presenting with severe symptoms, irrespective of age or underlying conditions. For cases presenting mild symptoms, antiviral treatment was recommended for high-risk patients only, which included infants <5 y, seniors >65 y, persons with lung disease (including asthma), cardiovascular disease (except for systemic arterial hypertension), renal disease, hematologic disease, neurologic disease, neuromuscular or metabolic disorders (including diabetes), and immune deficiency disease. Records with missing data were excluded from the analysis; less than 5% of records had one or more missing variables.

We defined the *admission delay* as the time elapsed from symptoms onset to hospitalization admission, and *hospital length of stay* was defined as the number of days from hospital admission to discharge or death. Given that recommendations for neuraminidase inhibitors stipulate that treatment should be provided within 48 h of disease onset, we stratified admission delay into two groups: <=2 and >2 days and assessed the association between admission delay, antiviral treatment, and disease severity. To evaluate potential differences in disease severity over time and between regions, we considered three temporally-distinct pandemic waves in the spring (April 1 to May 20), summer (May 21 to August 1) and fall (August 2 to December 31) of 2009 as in past work [7]. The spring pandemic wave was mainly confined to the greater Mexico City area and other central states; the summer wave focused on southeastern states; and the third wave was associated with widespread influenza activity.

### Case fatality ratios

The case fatality ratio (CFR) measures the proportion of deaths from all symptomatic infections, e..g, the probability that an infection causes death, and can be used as a measure of disease severity. Here we estimated the case fatality ratio among ILI cases, namely the proportion of ILI deaths among all ILI cases, which potentially includes the contribution of non-influenza pathogens (CFRili = ILI deaths/ILI cases). We also estimated the case fatality ratio among laboratory-confirmed A/H1N1 influenza outpatients and inpatients (CFRflu = A/H1N1 deaths/A/H1N1 cases), and the case fatality ratio among laboratory-confirmed A/H1N1 hospitalizations (CFRh = A/H1N1 inpatient deaths/A/H1N1 inpatient cases). Next, we analyzed the case fatality ratio according to neuraminidase inhibitor use, admission delay, age groups, pandemic waves, and geographic regions.

### Multivariate regression analysis

We used multivariate logistic regression to evaluate the risk of death among A/H1N1 inpatients after adjusting for age, gender, pandemic wave, geographic region, admission delay (<=2 days vs. >2 days), and antiviral treatment. The interaction between antiviral treatment and admission delay was also quantified due to the fact that patients admitted earlier in their disease course had a higher probability of receiving antiviral treatment. The effects of model predictors were measured using odds ratios (95% CI) and P values. Records with missing data (e.g., admission delay, antiviral use) were excluded from the analysis.

Statistical analyses were performed using PASW Statistics 18.0 and Matlab (The Mathworks, Inc).

Ethics Committee approval was not necessary according to local regulations. All the data were de-identified. Data employed in this study are routinely collected for epidemiological surveillance purposes.

## Results

### Temporal pandemic profile, admission delays, and length of hospital stay

**Figure 1:**
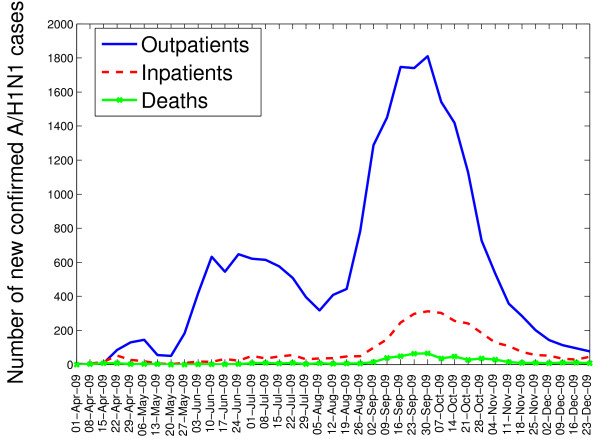
Weekly number of new laboratory-confirmed A/H1N1 influenza outpatients, inpatients, and deaths by date of symptoms onset from April 1 to December 31, 2009 in Mexico.

The characteristics of laboratory-confirmed A/H1N1 influenza cases by pandemic wave are shown in Table 1, and the temporal profile of outpatients, hospitalizations, and deaths in the IMSS database is illustrated in Figure 1. Overall, there were 117,818 ILI cases reported to the IMSS system between April and December 2009. The total number of laboratory-confirmed A/H1N1 cases (hospitalizations) reported in the spring, summer, and fall waves were 615 (159), 5,741 (371), and 21,081 (3,461), respectively. A similar proportion of females and males were affected during each of the three pandemic waves (Chi-square test, P = 0.91, Table 1).

**Table 1:** Characteristics of laboratory-confirmed A/H1N1 influenza cases by pandemic wave, Mexico, 01 April through 31 December, 2009

**Variable**	**Pandemic wave**			
	**Wave 1**	**Wave 2**	**Wave 3**	**P value**^**a**^
Geographic				
Central	433/615 (70.4)	602/5741 (10.5)	9948/21081 (47.2)	<0.0001
Southern	58/615 (9.4)	3734/5741 (65)	2322/21081(11)	
Other states	124/615 (20.2)	1405/5741 (24.5)	8811/21081 (41.8)	
Demography Female	310/612 (50.4)	2922/5690 (51)	10780/21173 (51.2)	0.91
Age (years)				
<18	277/611 (45)	3020/5688 (53)	10187/21167 (48)	<0.0001
18–49	283/611 (46)	2410/5688 (42)	9416/21167 (44)	
>50	51/611 (8)	258/5688 (5)	1564/21167 (7)	
Patients according to severity				
Outpatients	456/615 (74.2)	5370/5741 (93.5)	17620/21081 (83.6)	<0.0001
Hospitalizations	131/615 (21.3)	330/5741 (5.7)	2944/21081(14.0)	
Deaths	28/615 (4.6)	41/5741 (0.7)	517/21081 (2.4)	

Wave 1 (spring) refers to April 1 through May 20; wave 2(summer) refers to May 21 through August 1; wave 3 (fall) refers to August 2 through December 31 of 2009. Data are percentages of cases unless otherwise specified.

^a^ Determined by the Chi-square test statistic.

The average admission delay among laboratory-confirmed A/H1N1 inpatients was 3.1 days (95% CI: 3.0, 3.2). About half of the A/H1N1 inpatients in the spring wave were admitted within 2 days of symptoms onset (Table 2). The admission delay was longer on average during the spring than the summer and fall (4.2 d vs. 3.1 d, Wilcoxon test, P = 0.006) and lower in the southeastern region than in the rest of Mexico (2.5 d vs. 3.2 d, Chi-square test, P < 0.0001). The fraction of deaths among A/H1N1 inpatients was ~3-fold higher among those with admission delays >2 days compared to A/H1N1 inpatients with admission delays < =2 days (Chi-square test, P < 0.0001). Moreover, A/H1N1 inpatients who died in the hospital experienced longer admission delay than those who recovered (4.8 (95% CI: 4.4, 5.1) vs 2.8 days (95% CI: 2.7, 3.0), Wilcoxon test, P < 0.0001). The average admission delay was significantly shorter among persons <50 y (3.0 days (95% CI: 2.9, 3.1)) than in older individuals (3.7 days (95% CI: 3.4, 4.1)).

**Table 2:** Characteristics of laboratory-confirmed A/H1N1 influenza inpatients according to admission delay, Mexico, 01 April through 31 December, 2009

**Variable**	**Time from symptoms onset to admission**		**P value**^**a**^
**Days from symptoms onset to admission**	**Group 1 < =2 days**	**Group 2 > 2 days**	
**No. of patients (% of all study inpatients)**	2223 (58%)	1610 (42%)	P < 0.0001
**Disease outcome**			
Deaths, No. (%)	177(8%)	375 (23.3%)	P < 0.0001
**Pandemic wave**			
Spring wave	73 (50.3%)	72 (49.7%)	P = 0.085
Summer wave	223 (61.1%)	142 (38.9%)	
Fall wave	1927 (58.0%)	1396 (42.0%)	
**Gender**			
Females, No. (%)	1293 (58.2%)	889 (55.2%)	P = 0.07
**Age group (y), No. (% in each group)**			
< 18	800 (65.4%)	424 (34.6%)	P < 0.0001
18–49	1114 (56.5%)	857 (43.5%)	
>50	307 (48.3%)	328 (51.7%)	
Spring wave	31 (44.3%)	39 (55.7%)	P = 0.003
Summer wave	114 (64.4%)	63 (35.6%)	
Fall wave	197 (65.7%)	103 (34.3%)	

^a^ Chi-square test.

The average length of hospital stay among A/H1N1 inpatients was 5.3 days (95% CI: 5.1, 5.6) and did not change over time (P = 0.07). However, length of stay was significantly shorter among A/H1N1 inpatients with admission delays < =2 days (4.2 days (95% CI: 3.9, 4.4)) compared to inpatients with admission delays >2 days (5.9 days (95% CI: 5.5, 6.3), Wilcoxon test, P < 0.0001).

### Temporal and regional patterns of neuraminidase inhibitor administration

A total of 19,807 (16.8%) of ILI patients were treated with antivirals, with higher treatment proportion among laboratory-confirmed H1N1 patients overall (19.3% in A/H1N1-positive cases vs. 12.5% of A/H1N1-negative cases, Chi-square test, P < 0.0001). There was higher antiviral use in the outpatient than in the inpatient setting (20.1% vs. 14.1%, P < 0.0001, Table 3). Among A/H1N1 inpatients, antivirals were administered more frequently to patients with short admission delay (15.4% for delay < 2 dys vs. 12.7% for delays > = 2 days, Chi-square test, P = 0.021), with no difference between the spring and summer waves (P > 0.16).

**Table 3:** Rates of antiviral administration (mean and 95% confidence intervals) among laboratory-confirmed A/H1N1 influenza cases by pandemic wave, Mexico, 01 April through 31 December, 2009

**Variable**	**Total A/H1N1 cases**	**Pandemic wave**			**P value**^**a**^
		**Wave 1**	**Wave 2**	**Wave 3**	
No. patients that received antivirals (% of total A/H1N1 cases)	19.3% (18.8, 19.7)	48.6% (44.6,52.6)	54.9% (53.6,56.2)	8.7% (8.32,9.09)	P < 0.0001
Geographic					
Central	12.1% (11.5,12.7)	41.6% (36.9,46.4)	38.5% (34.6,42.6)	9.19% (8.63,9.78)	P < 0.0001
Southern	39.1% (37.9,40.4)	65.5% (51.9,77.5)	59.8% (58.3,61.4)	5.13% (4.27,6.11)	P < 0.0001
Other states	15.2% (14.5,15.9)	65.3% (56.3,73.6)	48.8% (46.2,51.5)	9.08% (8.48,9.7)	P < 0.0001
Female	19.4% (18.8,20.1)	47.7% (42.1,53.5)	53.8% (52,55.6)	9.33% (8.79,9.9)	P < 0.0001
Male	19% (18.4,19.7)	49.5% (43.8,55.3)	56.1% (54.2,57.9)	8.03% (7.51,8.57)	P < 0.0001
Age (years)					
<18	19.5% (18.9,20.2)	50.2% (44.2,56.2)	58.2% (56.5,60)	7.09% (6.59,7.6)	P < 0.0001
18–49	19.8% (19.1,20.5)	48.2% (42.3,54.2)	52.4% (50.4,54.4)	10.4% (9.79,11)	P < 0.0001
>50	14.2% (12.7,15.9)	43.1% (29.3,57.8)	40% (34,46.2)	8.99% (7.61,10.5)	P < 0.0001
Patients according to severity					
Outpatients	20.1% (19.6,20.7)	48.7% (44,53.4)	55.4% (54,56.7)	8.67% (8.25,9.09)	P < 0.0001
Inpatients	14.6% (13.4,15.8)	46.6% (37.8,55.5)	49.4% (43.9,54.9)	9.29% (8.26,10.4)	P < 0.0001
Deaths	11.1% (8.68,13.9)	57.1% (37.2,75.5)	39% (24.2,55.5)	6.4% (4.44,8.86)	P < 0.0001

Wave 1 (spring) refers to April 1 through May 20; wave 2 (summer) refers to May 21 through August 1; wave 3 (fall) refers to August 2 through December 31 of 2009.

^a^ Determined by the Chi-square test statistic.

**Figure 2:**
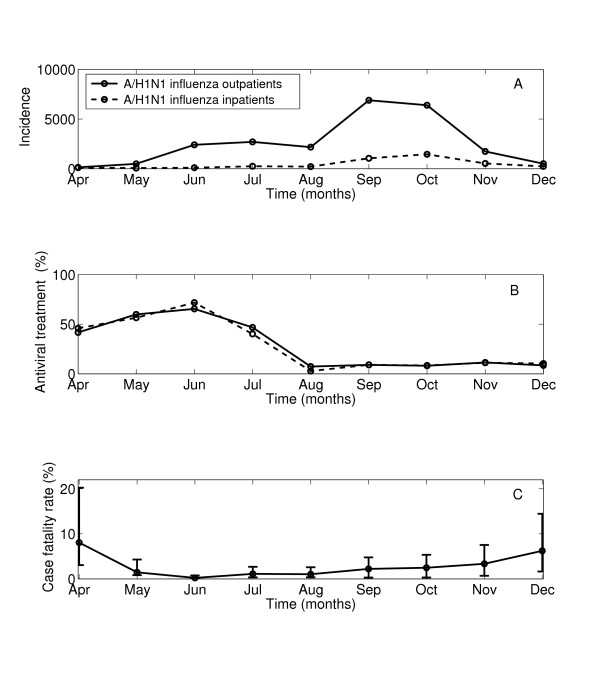
**Temporal evolution of neuraminidase inhibitor administration rates and the case fatality ratio by date of symptoms onset during the 2009 A/H1N1 influenza pandemic in Mexico, AprilDecember 2009.** A) Monthly number of new laboratory-confirmed A/H1N1 influenza outpatients and inpatients, B) Monthly percentage of laboratory-confirmed A/H1N1 influenza outpatients and A/H1N1 inpatients that received antiviral medications during the pandemic period, and C) the monthly case fatality ratio (and corresponding 95% CIs) based on all A/H1N1 influenza cases during the pandemic period.

There was a marked shift in patterns of antiviral administration at IMSS facilities by the fall of 2009. While antiviral administration remained high at 50% among H1N1-confirmed cases throughout the spring and summer pandemic waves (April-July, 2009) with peak use at ~70% in June, antiviral used declined significantly later in the pandemic to ~9% (Figure 2A-B and Table 3, Chi-square test, P < 0.0001). Southeastern states had 2.6-3.3 fold higher proportion of A/H1N1 influenza cases treated with neuraminidase inhibitors compared to other regions (Table 3).

No difference by gender in antiviral administration was observed among laboratory-confirmed A/H1N1 outpatients and inpatients (P = 0.38). Antiviral use was slightly lower among patients aged 50 years of age and older than among younger patients (Table 3, Chi-square test, P < 0.0001). Antiviral use declined with severity of disease, with treatment percentage of 20.1% among A/H1N1-positive outpatients, 14.6% among A/H1N1-positive inpatients, and 11.1% of A/H1N1-positive decedents (Chi-square test, P < 0.0001). Temporal trends of antiviral use were consistent by severity status, geographic region, gender, and age groups, and revealed consistently lower usage during the fall pandemic wave (Figure 2B, Table 3).

### Case fatality ratios

We found significant temporal and geographic differences in the influenza-related case fatality ratios (Figure 2C). The overall CFR was estimated at 2.1% (2.0, 2.3) based on laboratory-confirmed A/H1N1 cases and 1.2% (1.1,1.2) based on ILI cases. CFR estimates were significantly lower during the period of high antiviral use (April-July, CFRflu = 1.1%, CFRili = 0.9%) than during the period of low antiviral use in the fall (August-December, CFRflu = 2.4%, CFRili = 1.2%, Chi-square test, P < 0.0001, Table 4). This difference was even more pronounced after exclusion of the month of April associated with unusually long admission delays (May-July, CFRflu = 0.8%, CFRili = 0.6%, Chi-square tests, P < 0.0001).

**Table 4:** Case fatality ratios (mean and 95% CIs) among laboratory-confirmed A/H1N1 influenza cases by pandemic wave, Mexico, 01 April through 31 December, 2009

**Variable**	**Overall case fatality ratio**	**Pandemic wave**			**P value**^**a**^
		**Wave 1**	**Wave 2**	**Wave 3**	
Total A/H1N1 deaths (% of total A/H1N1 cases)	2.1% (2.0,2.3)	4.6% (3.1,6.5)	0.7% (0.5,1.0)	2.5% (2.3,2.7)	P < 0.0001
Central	2.7% (2.4,3.0)	5.5% (3.6,8.1)	1.7% (0.8,3.0)	2.7% (2.4,3.0)	P < 0.0001
Southern	0.8% (0.6,1.0)	0% (0,6.2)	0.5% (0.3,0.8)	1.2% (0.8,1.7)	P < 0.0001
Other states	2.3% (2.1,2.6)	3.2% (0.9,8.1)	0.9% (0.4,1.5)	2.6% (2.2,2.9)	P < 0.0001
Demography					
Female	2.1% (1.8,2.3)	4.5% (2.5,7.5)	0.6% (0.3,0.9)	2.4% (2.1,2.7)	P < 0.0001
Male	2.2% (2.0,2.5)	4.6% (2.5,7.6)	0.9% (0.6,1.3)	2.5% (2.2,2.8)	P < 0.0001
Age (years)					
<18	0.6% (0.5,0.8)	0.7% (0.1,2.6)	0.1% (0.04,0.3)	0.8% (0.6,0.97)	P < 0.0001
18–49	2.8% (2.5,3.1)	6.0% (3.5,9.4)	1.2% (0.8,1.7)	3.2% (2.8,3.6)	P < 0.0001
>50	8.5% (7.2,9.8)	15.7% (7.0,28.6)	3.5% (1.6,6.5)	9.1% (7.7,10.6)	P < 0.0001

Wave 1 (spring) refers to April 1 through May 20; wave 2 (summer) refers to May 21 through August 1; wave 3 (fall) refers to August 2 through December 31 of 2009.

^a^ Determined by the Chi-square test statistic.

Case-based CFR increased with older age, consistently across pandemic waves (Table 4). The CFR was lowest in the southeastern region, which also had the highest rates of antiviral administration and highest A/H1N1 influenza activity in summer (Table 3). Yet, the southeastern region also experienced a significantly lower CFRflu than other regions later in the pandemic, as antiviral use had decreased (1.16% vs. 2.61%, Chi-square test, P < 0.0001).

We also estimated the hospital-based CFR, CFRh, at 14.4% (13.3, 15.6). CFRh increased significantly with admission delay independently of geographic region, gender, and age groups (Table 5). The overall CFRh was about 3-fold higher for inpatients with admission delays >2 days than for those inpatients with admission delays < =2 days (Table 5). Consistent with geographical patterns in case-based CFR, CFRh was significantly lower in the southeastern region than in other regions (Table 5).

**Table 5:** Case fatality ratios (mean and 95% CIs) among laboratory-confirmed A/H1N1 inpatients according to admission delay, Mexico, 01 April through 31 December, 2009

**Variable**	**Overall case fatality ratio**	**Admission delay **		**P value**^**a**^
		**<=2 days**	**>2 days**	
Total A/H1N1 deaths (% of total A/H1N1 cases)	14.4% (13.3,15.6)	8.0% (6.9,9.17)	23.3% (21.2,25.4)	P < 0.0001
Geographic				
Central	14.9% (13.3,16.6)	7.9% (6.37,9.7)	23.9% (21,26.9)	P < 0.0001
Southern	7.2% (5.3,9.6)	4.0% (2.3,6.4)	13.5% (9.14,18.9)	P < 0.0001
Other states	16.9% (14.9,19)	10.1% (8.0,12.4)	26% (22.5,29.7)	P < 0.0001
Pandemic wave				
Spring	15.9% (10.3,22.8)	5.5% (1.5,13.4)	26.4% (16.7,38.1)	P = 0.001
Summer	10.4% (7.47,14)	5.8% (3.1,9.8)	17.6% (11.7,24.9)	P < 0.0001
Fall	14.8% (13.6,16)	8.3% (7.1,9.6)	23.7% (21.5,26)	P < 0.0001
Demography				
Female	12.5% (11.2,14)	7.4% (6.1,9.0)	19.9% (17.3,22.7)	P < 0.0001
Male	16.9% (15.1,18.8)	8.7% (7.0,10.7)	27.5% (24.2,30.9)	P < 0.0001
Age (years)				
<18	6.3% (5,7.8)	3.4% (2.2,4.9)	11.8% (8.9,15.3)	P < 0.0001
18–49	16.6% (15,18.4)	8.4% (6.9,10.2)	27.3% (24.3,30.4)	P < 0.0001
>50	23% (19.8,26.5)	17.9% (13.8,22.7)	27.7% (23,32.9)	P = 0.003

^a^ Determined by the linear Chi-square test statistic.

We found a consistent relationship between disease severity and antiviral use. Specifically, CFR was lower among treated than untreated patients if considering all confirmed A/H1N1 cases (1.2% vs. 2.3%, Chi-square test, P < 0.0001), all ILI cases (CFR = 0.8% vs. 1.2%, Chi-square test, P < 0.0001), or A/H1N1-positive inpatients alone (11.6% vs. 15.2%, Chi-square test, P = 0.03).

### Multivariate regression analysis

Antiviral treatment and hospital admission delays were significantly associated with the risk of death among A/H1N1 inpatients after adjusting for age, gender, geographic region, and pandemic wave in a multivariate logistic regression model with backward elimination. Antiviral treatment significantly reduced the risk of death (OR = 0.52 (95% CI: 0.30, 0.90)) while admission delays >2 days increased the risk of death by 2.8-fold (95% CI: 2.25, 3.41) among A/H1N1 inpatients. We included an interaction term between antiviral treatment and admission delay, which was statistically significant (OR = 2.0 (95% CI: 1.03, 3.85)), suggesting that the protective effect of treatment was negated among patients experiencing long admission delays. For the group of A/H1N1 inpatients with admission delays < =2 days, antiviral treatment significantly reduced the risk of death (OR = 0.53 (95% CI: 0.30, 0.94)) whereas for the group of A/H1N1 inpatients with admission delays >2 days, antiviral treatment did not significantly reduce the risk of death (OR = 0.96 (95% CI: 0.65, 1.43)).

Male inpatients experienced an increased risk of influenza-related death (OR = 1.46 (95% CI: 1.21, 1.77)) compared to females. Moreover, the southeastern region experienced a significant reduced risk of death compared to other regions (OR = 0.50 (95% CI: 0.35, 0.68)). The pandemic wave indicator was the only predictor that was eliminated from the model by the backward elimination procedure (P = 0.53).

## Discussion

We have carried out a detailed analysis of the association between case fatality ratio, admission delays, and neuraminidase inhibitor administration patterns in ILI and laboratory-confirmed pandemic influenza A/H1N1 patients. Our data are based on individual-level patient information collected through a prospective influenza surveillance system during Apr-Dec 2009 in Mexico. In our large sample comprising 117,818 patients, the risk of death among A/H1N1 inpatients was significantly associated with admission delay and antiviral treatment, in line with previous studies [2,11-17]. We also found that age, gender, and geography were significantly associated with risk of death. Our findings suggest significant antiviral effectiveness when administered during the early symptomatic phase (<=2 days) when antiviral treatment is considered clinically meaningful [18-21]. Our results also confirm an increasing case fatality ratio with older age [7,22,23]. Finally, our results reveal marked temporal trends in patterns of antiviral use in Mexico, dropping to ~9% by fall 2009 from 50% in earlier pandemic months.

We found the case fatality ratio based on laboratory-confirmed A/H1N1 influenza cases (outpatients and inpatients) to be 2- to 3-fold lower during the period of high antiviral administration during April-July, 2009 compared to the period of low antiviral administration (August-December, 2009). Accordingly, CFR was 1.5 to 1.9 times lower among ILI and A/H1N1 patients treated with antivirals compared to untreated patients [2,14]. For A/H1N1-positive inpatients with admission delay < =2 days, CFR was twice lower for the treated group than the untreated group, which is in agreement with previous studies [15-17].

We did not find any difference in length of hospital stay by antiviral treatment status. A recent study found that antiviral treatment reduced the length of stay by 18% for hospitalized children with seasonal influenza [21]. No pandemic study is available for comparison with our data.

We did not find evidence of temporal trends in severity of pandemic infections beyond those associated with antiviral use and hospital admission delay, as suggested by the lack of significance of the pandemic wave indicator in our multivariate model. This suggests that there was no meaningful change in severity of circulating pandemic influenza viruses throughout 2009 in Mexico, and resistance to antivirals was not an issue. This is in line with an analysis of the risk of death among hospitalized cases during the summer and fall 2009 A/H1N1 pandemic waves in England [24].

Hospital admission delay was the strongest predictor of death among laboratory-confirmed A/H1N1 influenza inpatients, followed by antiviral treatment, in a multivariate model adjusting for age, gender, and geographic region. Admission delays <2 days significantly decreased risk of death by 55-71% while antiviral treatment reduced risk of death by ~48%, consistent with previous studies [2,4,11-17,25-28].

Our multivariate logistic regression analysis also supported a ~46% (95% CI 21-77%) increased mortality risk of death among A/H1N1 male inpatients compared to females. One study has reported a higher risk of hospitalization among males with pandemic A/H1N1 influenza in South Korea [29]. Our results also indicated that the southeastern region experienced a reduced risk of A/H1N1 inpatient death after controlling for antiviral use and other factors. We do not expect that socio-economic or ethnic differences played a role given that all patients covered through the IMSS health system in Mexico are workers and their families. Overall this suggests that factors beyond admission delays and antiviral treatment could have played a role in the severity of the 2009 A/H1N1 influenza pandemic, including behavioral factors and case management practices. We were not able to ascertain whether the availability of critical case management and intensive care units differed by geographical area or over time.

The most intriguing finding of our study was perhaps the sharp drop in antiviral use from 50% in the spring and summer wave to 9% in the main fall pandemic in Mexico. Antiviral use was similar among inpatients and outpatients in the fall, suggesting that decision to treat was not related to symptoms severity. This pronounced change in clinical practice could be explained in part by the great uncertainty surrounding pandemic severity in the early pandemic stages, with early data suggesting atypically severe disease [30,31]. By the end of the summer 2009 pandemic wave, it became clear that the severity of the 2009 pandemic virus was comparable to that of contemporaneous seasonal influenza epidemics [32]. Accordingly, antivirals were administered much more conservatively although antiviral availability was not an issue at IMSS facilities. Overall, our findings suggest that higher rates of timely antiviral treatment during the 2009 fall pandemic wave in Mexico could have led to a substantially lower death toll. As the novel pandemic A/H1N1 virus continues to circulate around the world, antiviral treatment should be considered for the great majority of severe ILI patients requiring hospitalization.

Case fatality ratio estimates for the 2009 A/H1N1 influenza pandemic have differed by one to two orders of magnitude between countries [33-40]. Our case-based severity estimates for Mexico are relatively high at 2% for A/H1N1-positive cases and 14% for hospitalized A/H1N1-positive patients. These high estimates likely reflect a bias of the Mexican IMSS influenza surveillance system towards the higher levels of the severity pyramid due to difficulties in identifying asymptomatic or mild cases [35]. In general it is difficult to compare CFR estimates between countries due to differences in patient care and probability of seeking care. However, estimates of case fatality ratio are useful comparative measures of severity across regions of the same country, pandemic waves, age groups, and patterns of neuraminidase inhibitor administration.

Lower CFR estimates in other countries could reflect in part higher rates of neuraminidase inhibitor treatment and prophylaxis, compared to those in Mexico [41-44]. In the US, rates of antiviral treatment among hospitalized A/H1N1 patients remained high throughout the pandemic at 50-82% [4,45,46]. In contrast, the distribution of admission delays in our study is generally similar to that in other studies [13,44,47,48]. One study reported a short median admission delay of one day in emergency departments, antiviral treatment rates >99%, and no pandemic-associated death in an upper middle to high socioeconomic population in Chile [42].

Several strengths and caveats of our study are worth noting. We used data on ILI and laboratory-confirmed influenza cases reported to the IMSS, where about one-third of all ILI cases were consistently tested for influenza in all regions and throughout the main pandemic period [7]. We compared the case fatality ratio across regions, pandemic waves, age groups, and patterns of neuraminidase inhibitor administration in Mexico. Of note, there was no evidence of weaker disease surveillance in smaller states, and testing rates for novel A/H1N1 influenza remained stable throughout the pandemic period [7]. Moreover, only a small fraction of the records had missing data. Specifically, 0.2% of all records missing the date of symptoms onset, 4% of inpatient records missing admission delay, and antiviral treatment was missing in 0.1% of records. We also note that the exact timing of start of antiviral treatment was not available. Data on antiviral treatment was available for those patients treated upon initial consultation or admission in hospitals, but were not able to ascertain antiviral treatment for patients well initially and therefore untreated, and deteriorated subsequently during hospitalization. The study patients, who visited the IMSS facilities, were likely to be representative of those patients with more severe disease, given the representativeness of the IMSS system. We note that antiviral treatment patterns among IMSS-affiliated populations may have been slightly higher than among non-IMSS populations, given the slightly higher socio-economic status of IMSS affiliates. Further, due to lack of information on the sensitivity and specificity of the ILI and ARI definitions used in the IMSS system [49], we considered both ILI/ARI and laboratory-confirmed outcomes in our analyses.

Another limitation of our analysis of disease severity was to disregard the impact of underlying chronic conditions. Our study was focused on relative comparisons of disease severity over time, geography, and treatment groups, and we assumed similar underlying conditions in comparison groups. Lastly, a subset of our A/H1N1-positive patients may have had secondary bacterial infection, but no data was available to evaluate the role of bacterial coinfections or antibiotic treatment on severity of A/H1N1 infection [50,51].

## Conclusions

We found an association between the case fatality ratio of the 2009 A/H1N1 influenza pandemic in Mexico and hospital admission delays, neuraminidase inhibitor treatment, demographics, and geography. Our results suggest that differences in antiviral treatment rates and admission delays could partly explain the reported variability in pandemic mortality burden between high and middle-income countries. Severity patterns in low-income regions, especially in Africa, may differ from those reported here and be related to frequency of underlying chronic conditions or access to care. More information on disease severity in low-income regions is warranted. Overall, our study underscores the potential impact of decreasing admission delays and increasing antiviral use in the inpatient setting to mitigate the mortality burden of future influenza pandemics.

## Competing interests

LS reports to have received consulting fees from IMS (a data analytics business), and from BioCryst, and served on an advisory board for Genentech.

## Authors’ contributions

GC, CV, LS and VHB designed the study. SEZ, MGL and VHB participated in data acquisition. GC analyzed the data and wrote the first draft of the manuscript. GC, CV, LS and VHB participated in the interpretation of results. GC, CV, LS, MM, MGL, SEZ, VHB contributed to the writing and editing of the manuscript. All authors read and approved the final manuscript.

## Pre-publication history

The pre-publication history for this paper can be accessed here:

http://www.biomedcentral.com/1471-2334/12/97/prepub
